# A High Performance Torque Sensor for Milling Based on a Piezoresistive MEMS Strain Gauge

**DOI:** 10.3390/s16040513

**Published:** 2016-04-09

**Authors:** Yafei Qin, Yulong Zhao, Yingxue Li, You Zhao, Peng Wang

**Affiliations:** The State Key Laboratory for Manufacturing Systems Engineering, Xi’an Jiaotong University, No. 28, Xianning West Road, Xi’an 710049, China; yafeiqin@stu.xjtu.edu.cn (Y.Q.); yingxueli@stu.xjtu.edu.cn (Y.L.); zhaoyou319@stu.xjtu.edu.cn (Y.Z.); wangpeng1851@163.com (P.W.)

**Keywords:** torque, sensitivity, frequency, MEMS strain gauge

## Abstract

In high speed and high precision machining applications, it is important to monitor the machining process in order to ensure high product quality. For this purpose, it is essential to develop a dynamometer with high sensitivity and high natural frequency which is suited to these conditions. This paper describes the design, calibration and performance of a milling torque sensor based on piezoresistive MEMS strain. A detailed design study is carried out to optimize the two mutually-contradictory indicators sensitivity and natural frequency. The developed torque sensor principally consists of a thin-walled cylinder, and a piezoresistive MEMS strain gauge bonded on the surface of the sensing element where the shear strain is maximum. The strain gauge includes eight piezoresistances and four are connected in a full Wheatstone circuit bridge, which is used to measure the applied torque force during machining procedures. Experimental static calibration results show that the sensitivity of torque sensor has been improved to 0.13 mv/Nm. A modal impact test indicates that the natural frequency of torque sensor reaches 1216 Hz, which is suitable for high speed machining processes. The dynamic test results indicate that the developed torque sensor is stable and practical for monitoring the milling process.

## 1. Introduction

In recent years, high speed machining become more and more important because of its advantages such as high production efficiency, high machining accuracy, long life of cutting tools and so on. In high speed machining processes, the cutting force directly affects the mechanical quality of the workpiece. Cutting force measurements are also an essential requirement to monitor manufacturing processes and they offer an important indicator to design machine tools, optimize machining processes, predict the surface roughness, improve the accuracy of the workpiece and detect machining tool vibrations, monitor machining cutter wear and so on. Many examples show that the cutting parameters such as cutting speed, feed rate and cutting depth often have certain deviations from the initial settings during cutting processes. It is necessary to measure the cutting force experimentally because accurate theoretical cutting force calculations cannot be performed due to the complex cutting conditions which have unsure stresses and factors. For this purpose, many dynamometers have been designed and manufactured, which are mainly based on elastic deformation of materials and used for monitoring turning and milling operations.

In milling and drilling processes a table dynamometer is often typically used for monitoring the cutting force. Yaldiz *et al.* [1] developed a table dynamometer to measure static and dynamic cutting forces based on a strain gauge. It can measure three perpendicular cutting force components and torque. Differently, Korkut [2] also developed a three-force component dynamometer which is composed of four octagonal rings placed in-house between two plates and fixed with screws. Scheer *et al.* [3] developed a spindle-integrated force sensor based on a piezoelectric ring for milling and drilling. Byrne *et al.* [4] and Park *et al.* [5] did a similar study with a piezoelectric force ring installed into the spindle flange, where the data could be obtained through a stator via telemetry from the rotating sensor. Totis *et al.* [6] also developed a rotating dynamometer to measure triaxial cutting force components in face milling. In the past, some works on spindle-integrated force sensors and rotating dynamometers based on strain gauges have been reported, and these devices could sense elemental elastic deformations with high sensitivity and output a corresponding voltage. For example, Smith *et al.* [7] and Suprock *et al.* [8] proposed a sensor integrated spindle for torque measurement; similarly, Adolfsson and Stahl [9] developed a dynamometer for measuring cutting force components at each cutting edge for face milling. Rizal *et al.* [10] developed an integrated rotating dynamometer based on a strain gauge which measured three cutting force components and was assembled with variety of cutting tools by the flange. There are some defects in previous research on either piezoelectric sensors or strain gauge force sensors. For piezoelectric sensors, electric charge leakage may happen during high speed milling processes and commercial piezoelectric force dynamometers (e.g., the Kistler 9257B) cost too much, even though they offer good advantages of high sensitivity, stiffness and resonant frequency. Strain gauge milling force sensors must deal with conflicting characteristics like sensitivity and natural frequency so one of them must be sacrificed in actual industrial applications.

Milling torque force is recognized as a good feature for online chatter detection because of its close relationship to the material removal mechanism and its independence from the effects of tool path relative to the workpiece [11]. According to this characteristic, this paper presents a study describing an integrated torque sensor using a piezoresistive MEMS strain gauge which has small size and highly sensitivity. Polymer and silicon were usually used as materials of MEMS sensors and actuators. Liu [12] provided a comprehensive review of polymer-based MEMS, including materials, fabrication processes, and representative devices. It indicated that many polymer materials had excellent advantages for use in MEMS such as greater mechanical yield strain, significantly lower cost, being easily obtained, unique chemical, structural and biological functionalities and so on. For example, Kottapalli *et al.* [13,14] developed a reliable, robust, low fabrication cost and highly sensitive pressure sensor, which employed a liquid crystal polymer as a structural material. This demonstrated that polymer materials were a good choice to develop MEMS sensors for complex environments. The torque force sensing element proposed in this research is an analogue hollow cylinder structure bonded with a piezoresistive MEMS strain gauge. The MEMS strain gauge employs silicon as the substrate and is fabricated by Silicon-On-Insulator (SOI) technology, metal wires placed on the substrate are connected to a Wheatstone bridge, and the output voltage signal is transmitted by a wireless network during the milling process. This paper outlines the design and fabrication of the torque sensor, fabrication of the piezoresistive MEMS strain gauge, a method for bonding the strain gauge onto the sensing element, static calibration, model impact and dynamic tests. Experimental results indicate that the torque sensor has high performance with high sensitivity and natural frequency. The sensor developed in this work solved the principal contradiction between sensitivity and natural frequency, and the milling test results also demonstrate that the torque sensor based-MEMS strain gauge could be used to detect the stability and accuracy of the machine tool for real-time applications.

## 2. Design and Fabrication

### 2.1. Design Principle

**Figure 1:**
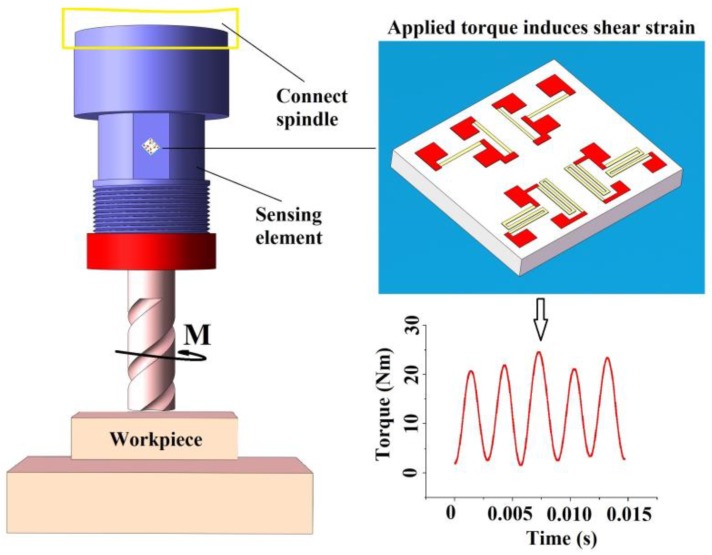
A diagram of the torque sensor using a piezoresistive MEMS strain gauge for measuring torque force. The MEMS strain gauge is 1.8 mm × 1.6 mm × 0.2 mm. The strain gauge detects shear strain with any torque force applied in a milling process.

The purpose of this study was to develop a high performance sensor using piezoresistive MEMS strain gauges which can monitor milling torque forces during machining processes in real-time, as shown in Figure 1. The shear strain of the strain gauge will change when a torque force is applied to the sensing element. As a critical component, the sensing element influences the sensitivity and other properties of the whole sensor to a large extent. In this study, a thin-walled cylinder was chosen as sensing element, as illustrated in Figure 1, and when only torque force is applied to it, the principle strains *ε*_x_ and *ε*_y_ are tiny and we can ignore them in the calculations, thus the main shear strain transformation can be written as Equation (1) under the present circumstances. According to mechanical common sense, it is obvious that the maximum shear strain is produced when the angle *θ* is ±45°, as shown in Equation (2):

(1) $$\varepsilon_{T}(\theta )=\gamma_{xy}\sin\theta \cos\theta$$

(2) $$\gamma_{\left(45^{\circ }\right)}=\frac{T}{2\pi Gtr^{2}}$$

where *T* = torque force applied to sensing element, *G* = shear modulus of rigidity, *r* = the outer radius of thin-walled cylinder, and *t* = the thickness of the sensing element.

From Equation (2), it is observed that the shear strain will be greatly decreased with any slight increase of the radius under a certain torque value. The measuring sensitivity is directly proportional to the shear strain sensitivity, which will be reduced with the increase of the design parameters *r* and *G*.

For a torque measurement sensor, both high stiffness and high sensitivity mean high performance, so it should be easy to determine which design parameters can affect these properties. The strain sensing element can be modeled as a spring element with appropriate stiffness. Equation (3) shows the torsional stiffness *K_ms_* of the strain sensing element, where *G*, *t* and *r* are defined in Equation (2), and *L* is the length of the thin-walled cylinder.

(3) $$K_{ms}=\frac{2\pi Gtr^{3}}{L}$$

**Figure 2:**
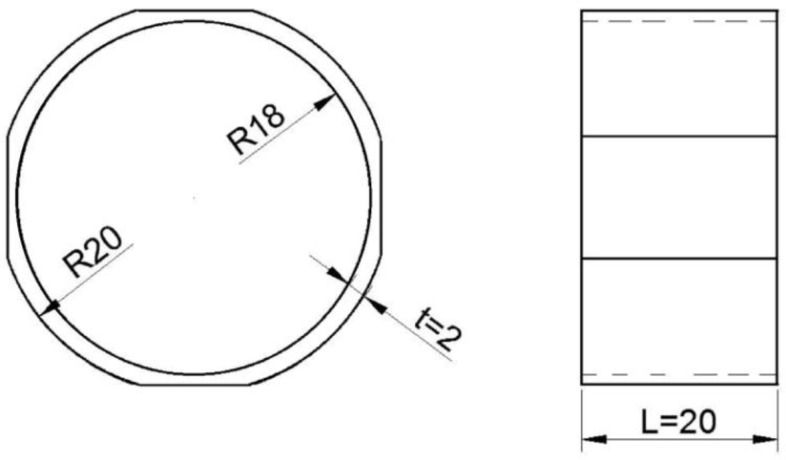
Schematic view of the thin-walled cylinder.

Equations (2) and (3) indicate that there are three design parameters *t*, *r* and *L* which must be specified for the sensing element. Through comparison of Equations (2) and (3), it is obvious that the sensing element length *L* should be minimized because it will increase the stiffness without affecting the shear strain sensitivity. The stiffness equation shows a cube dependance on *r* compared with its quadratic dependance in the shear strain sensitivity equation. This indicates that the radius parameter *r* should be maximized in order to increase the stiffness while the thickness *t* should be as small as possible in order to increase the sensitivity. Of course, neither the radius of the sensing element should not be so large as to interfere with the milling operations nor the thickness too small to prevent buckling or damage. With comprehensive analysis of the above factors, the optimal sizes of the sensing elements are shown in Figure 2, where the outer radius, thickness, and length are 20 mm, 2 mm and 20 mm, respectively. For convenience of bonding with the strain gauge on the surface, four small platforms were fabricated by a machining operation.

**Figure 3:**
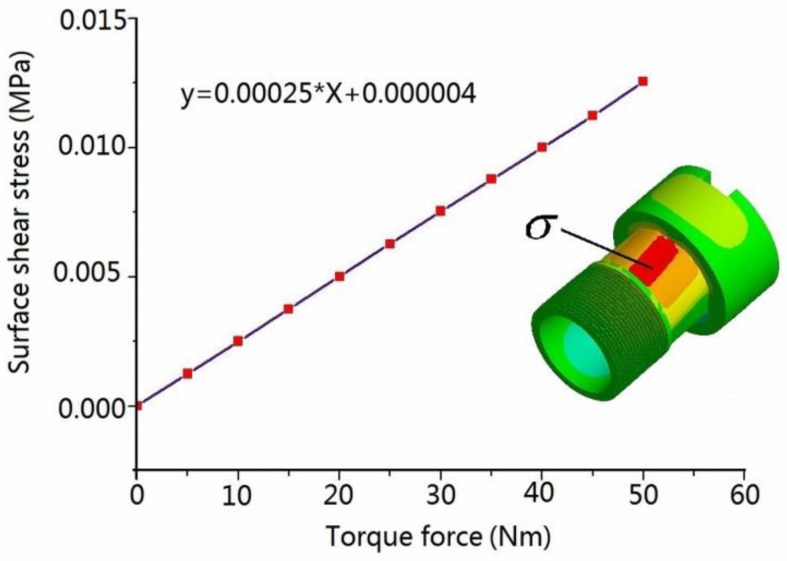
FEM result for the thin-walled cylinder under torque force.

In this work, 17-4PH stainless steel was chosen as sensing element material, whose elastic modulus and Poisson ratio are 2.1 GPa and 0.269, respectively. The surface stress of the thin walled cylinder was estimated through FEM simulation because it is difficult to obtain from a formula. The mesh element in this model is 2 mm. Figure 3 shows the variation of stress when the thin walled cylinder is under linear torque force. As can be seen, the outer surface stress has a good linear relationship with the applied torque force, which demonstrates that thin walled cylinder is suitable as a sensing element for this study. For a thin walled cylinder under the torque force *T*, the maximum stress change is distributed along ±45° referring to the central axis, and one was positive and the other was negative. This peculiarity could be well utilized to measure stress using a Wheatstone circuit bridge.

### 2.2. Sensor Design

**Figure 4:**
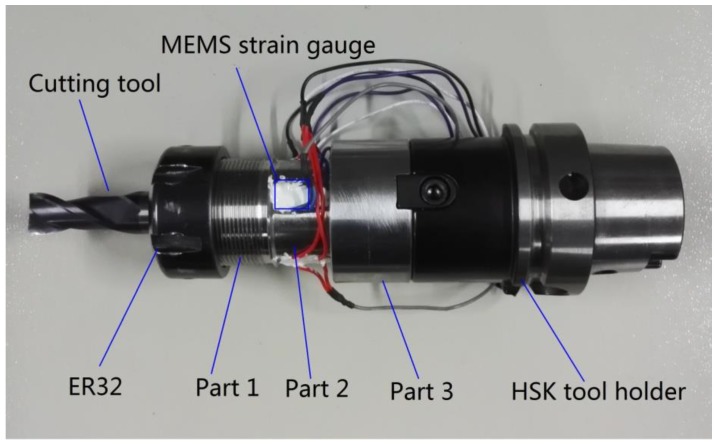
Photograph of the fabricated torque sensor.

This study developed a high sensitivity and high natural frequency torque force sensor for milling operations as depicted in Figure 4, which consists of three parts: Part 1 is a standard interface in order to fix the different types of milling cutters by a collet chuck named ER32; Part 2 is the thin walled cylinder used as sensing element with a bonded piezoresistive MEMS strain gauge which senses the stress and translates it into a voltage signal; and the third part is a standard interface connecting with a HSK tool holder.

**Figure 5:**
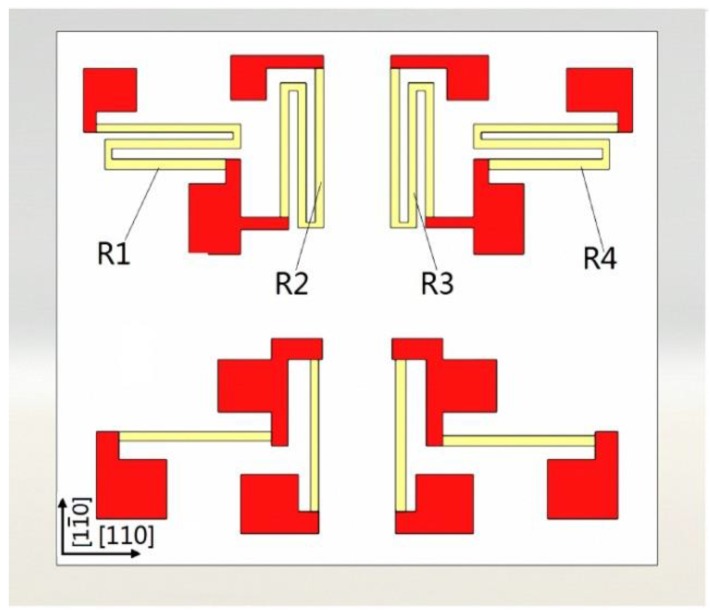
Schematic view of the designed MEMS strain gauge.

When voltage is applied to the piezoresistance to carry out dynamic monitoring in a machining process, it is possible that the PN junction may produce a leakage current, and thus affect the measurement accuracy and performance of the sensor. In order to solve this problem, Silicon-On-Insulator (SOI) was chosen as the material to fabricate our piezoresistive MEMS strain gauge. The structure of the piezoresistive MEMS strain gauge adopted in this study is shown in Figure 5. It consists of eight metal wire resistances, with half of them (1 KΩ each one) of the top and the remaining ones underneath (333 Ω each one). Taking the top as an example, four resistances can be used to organize an independent Wheatstone circuit bridge, where resistances R1 and R4 along the the [110] direction and R2 and R3 in the [$1\bar{1}0$] direction are included. Due to the excellent mechanical and electrical properties of the [100] crystal plane of monocrystalline silicon, a piezoresistive strain gauge whose size is 1.8 mm × 1.6 mm × 0.2 mm has been fabricated by MEMS technology.

**Figure 6:**
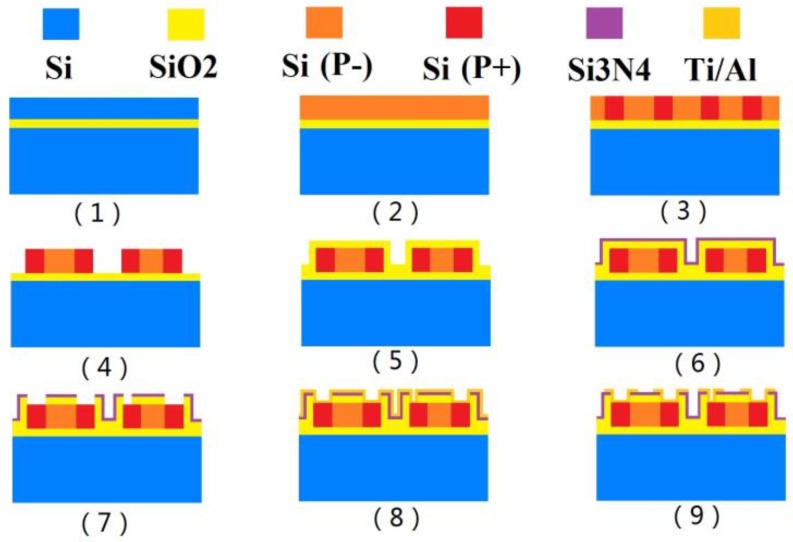
Fabrication process of MEMS strain gauge.

**Figure 7:**
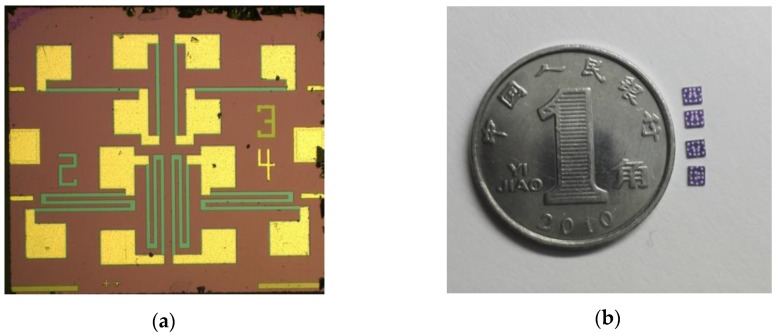
(**a**) SEM of MEMS strain gauge; (**b**) a photograph of MEMS strain gauge.

The piezoresistive MEMS strain gauge was processed mainly through the following steps as shown in Figure 6. First, a Silicon-On-Insulator wafer is cleaned with hydrogen fluoride solution; second, we performed p-type boron ion doping on the SOI wafer surface with an ion implantation system. After that, an annealing treatment in a 1100 °C nitrogen environment is maintained for one hour to distribute the mixed concentration of boron ion evenly on the surface; then, we carry out positive photolithography and dense ion implantation of boron for the ohmic contact area in order to form a low resistance ohmic contact area; in the fourth step, the remaining Si areas are etched to form the embossed resistance and graphics of the ohmic contact area on both sides of the resistance; fifthly, we obtain SiO_2_ thin films by using a thermal oxidation process; sixthly, using low pressure chemical vapor deposition (LPCVD) Si_3_N_4_ thin films are formed; seventh, positive photolithography is performed so as to form wire holes between the aluminium wire and resistance; eighth, we obtain a Ti/Al layer by physical vapor deposition on the positive silicon wafer and then form the metal electrode leading wire tunnel by Inductively Coupled Plasma (ICP); finally, using ICP etching, useless metal is removed. Figure 7a shows a SEM photograph of the MEMS strain gauge, and some as-fabricated strain gauges are presented in Figure 7b.

### 2.3. Bonding

Bonding is another critical aspect which strongly influences the output sensitivity of piezoresistive MEMS strain gauges. Several successful bonding methods based on silicon on the surface of stainless steel substrates were proposed [15,16,17,18,19].

The piezoresistive MEMS strain gauge may rotate away from the original direction or tilt [20] which could cause strain measurement errors. Bonding directions could be controlled reasonably by marking out the positioning line on the surface of sensing element. In addition, highly technical requirements in manufacturing sensing elements such as flatness, roughness could ensure the steel surface is clean and flat. In this case, the strain measurement sensitivity errors caused by the tilt are expected to be reduced to a minimal impact.

The piezoresistive MEMS strain gauge and PCB with a circular through-hole in the centre were bonded to the stainless steel surface using M-Bond 610 epoxy, which is a solvent-thinned, epoxy-phenolic adhesive for high-performance applications. As a first step, the gauging area was thoroughly degreased with GC-6 isopropyl alcohol, and a ballpoint pen was used to draw alignment marks on the sensing element surface in order to make sure that strain gauge is installed in the right place, then it was cleaned with ethyl alcohol and wiped dry with a gauze sponge. Secondly, M-Bond 610 was applied to the sensing element surface bonding area and the back of the strain gauge and PCB with a circular through-hole in centre, and the assembly was set each aside to air-dry for at least 15 min. Then the piezoresistive MEMS strain gauge and PCB assembly was returned to its original position over the layout marks. The assembly of strain gauge and PCB was tacked down with a certain amount of pressure. Spring clamps were used to apply a pressure of about 200 kN/m^2^ during the curing cycle. Then the clamped sensor was place into an oven and the temperature raised to 125 °C at a rate of approximately 10 °C per minute and held there for 2 h. Step 2 must be completed within 4 h. After remaining for 2 h in 125 °C, the sensor should be naturally cooled to room temperature. Finally, the sensor is placed in the oven and the temperature raised to 160 °C and maintained 2 h and then cooled to room temperature in a natural environment repeatedly. The bonding and curing treatment are completed by these steps.

## 3. Static Calibration

**Figure 8:**
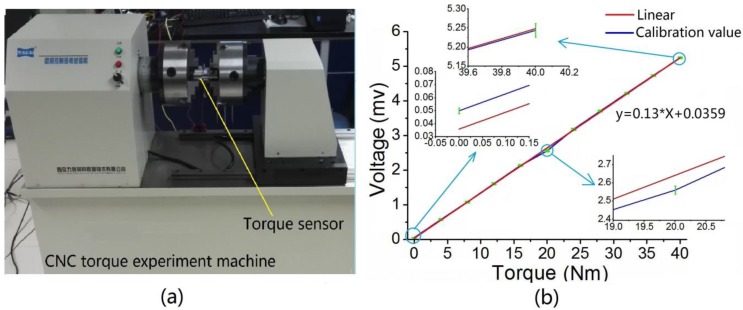
(**a**) Experimental setup for static calibration; (**b**) Calibration curve for the torque force.

In this work, the testing apparatus used to measure the stress are shown in Figure 8a. They include a CNC testing machine (WNZ-200, XI’AN LETRY, Xi’an, China) that could provide constant torque force, a digital multimeter (8846A, FLUKE CORPORATION, Everett, WA, USA) which could gauge voltage and a power supply (GPS-3303C, GWINSTEK Electronic Technology Co. Ltd, Suzhou, China) which could apply an excitation voltage of 5V DC for the torque sensor. To operate the testing apparatus, a torque force was applied to the end face by the electronic force regulator. The measurement range of the torque force is expected to be 0–40 Nm. The applied torque force was raised from zero to its maximum value in steps of 4 Nm, and holding time was maintained for 20 to 40 s in each step. The measuring circuits were excited by the power supply and the output voltage were recorded by the high-accuracy and high-resolution digital multimeter. Five cycles including loading and unloading procedures were implemented, and calibration result of the torque force is depicted in Figure 8b. As shown in the figure, the calibration data have been described with a red solid line obtained by linear fitting and error bars. Three calibration values with error bars were taken as examples, and respectively partially magnified. The experimental sensitivity of the torque sensor voltage signal (un-amplified) is 0.13 mv/Nm. The developed sensor possesses favorable static properties with a linearity error of less than 1.6%, which implies that it can meet the goal of high accuracy measurement.

## 4. Model Impact Test

**Figure 9:**
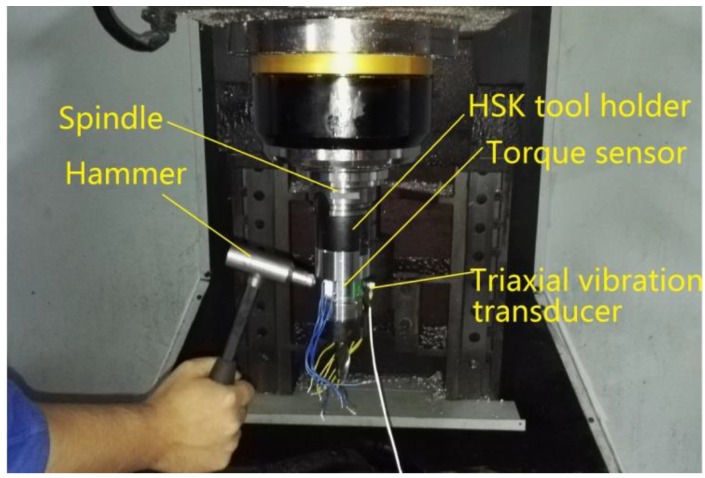
Experimental setup of the modal impact tests.

**Figure 10:**
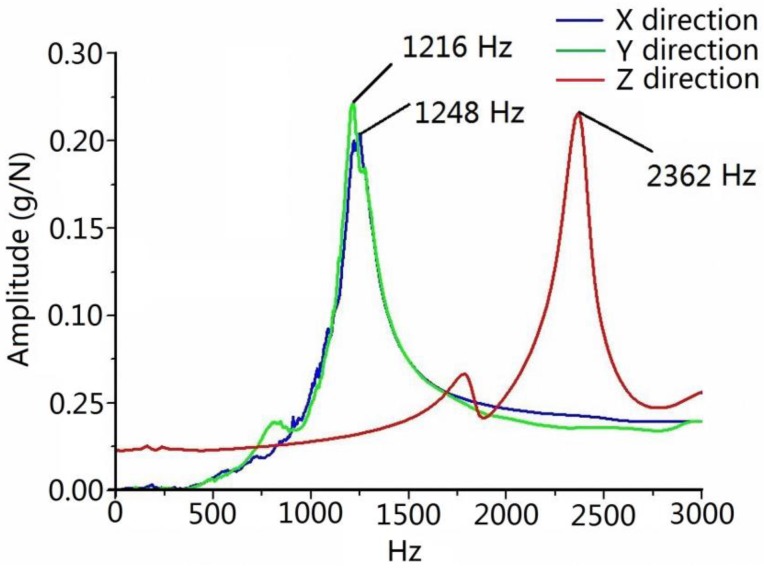
Frequency and amplitude result of the impacting modal test.

The system response of the sensor reflects its dynamic performance, which needs to be considered when the torque sensor is installed on the spindle monitoring machine tool in the cutting process. The natural frequency of the sensor affects the dynamic response of the system, which must be much higher than the frequency produced by the cutting tool in order to ensure that the collected signals would not be disturbed during the cutting process. Generally, the natural frequency should be at least four times larger than the frequency caused by the cutting tool. To identify the natural frequency of the torque sensor under actual working conditions, the natural frequency of the torque sensor system was obtained by a modal impcting test. The torque sensor was excited by a modal impact hammer (type 086D05), and a triaxial vibration transducer (type 95663) was connected to the sensing element, as illustrated in Figure 9. The signals excited by hammer and vibration transducer were acquired by a data acquisition and modal analysis system (type SCADAS305) manufactured by LMS Company (Leuven, Belgium). Finally, the natural frequencies of the sensor in three directions were calculated by the LMS Test Lab software. Figure 10 shows the amplitude-frequency functions in three directions. It is obvious that the natural frequencies of the sensor are approximately 1216, 1248 and 2362 Hz. In this study, cutting tests were performed using a two toothed end mill. On account of the lowest natural frequency of 1216 Hz, this sensor can be used for dynamic cutting force measurements below 1126/4 × ½ × 60 = 8445 rpm. If a four toothed end mill were chosen as cutting tool, the spindle speed would be limited to 4220 rpm in the actual machining process.

## 5. Cutting Test and Discussion

**Figure 11:**
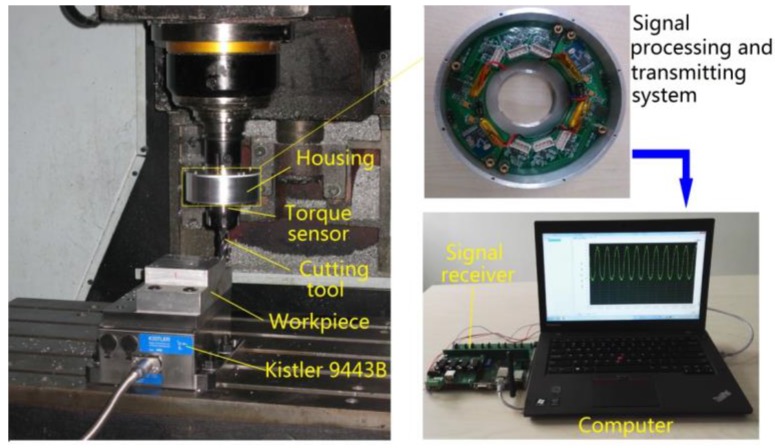
Dynamic cutting experiment.

**Figure 12:**
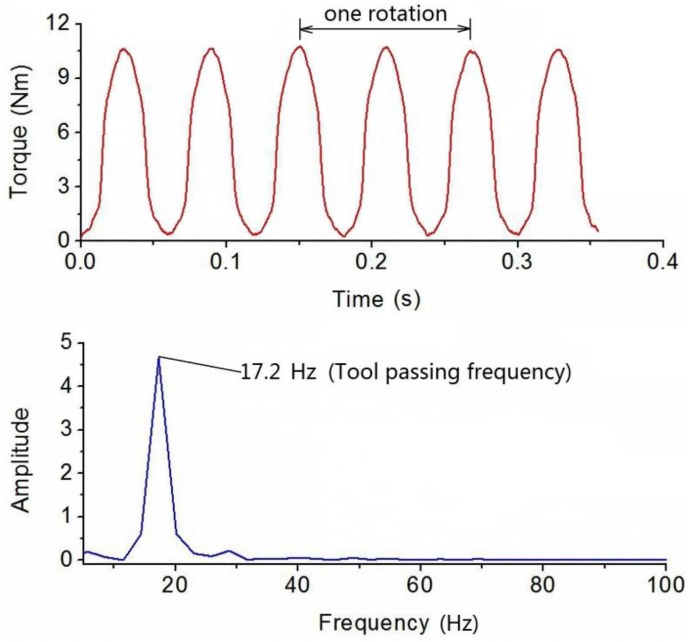
Plots of torque force signals in time and frequency domain at a spindle speed of 500 rpm.

**Figure 13:**
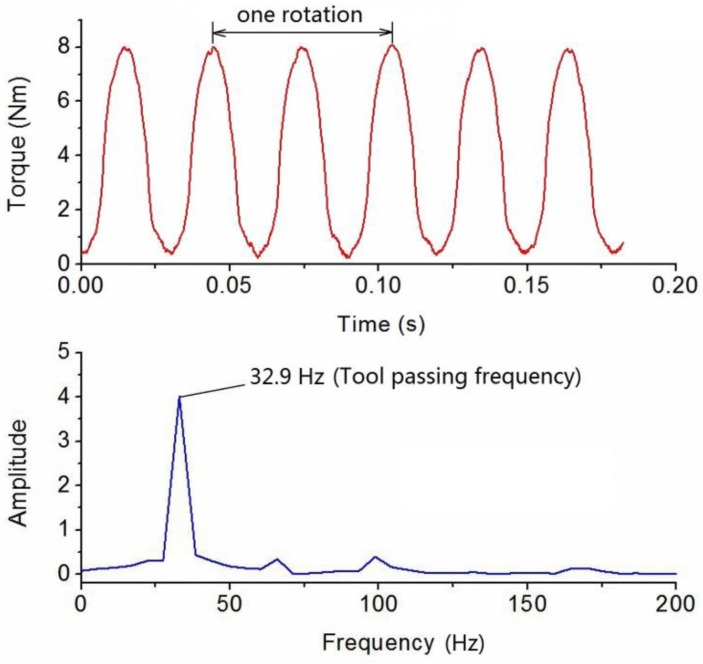
Plots of torque force signals in time and frequency domain at a spindle speed of 1000 rpm.

**Figure 14:**
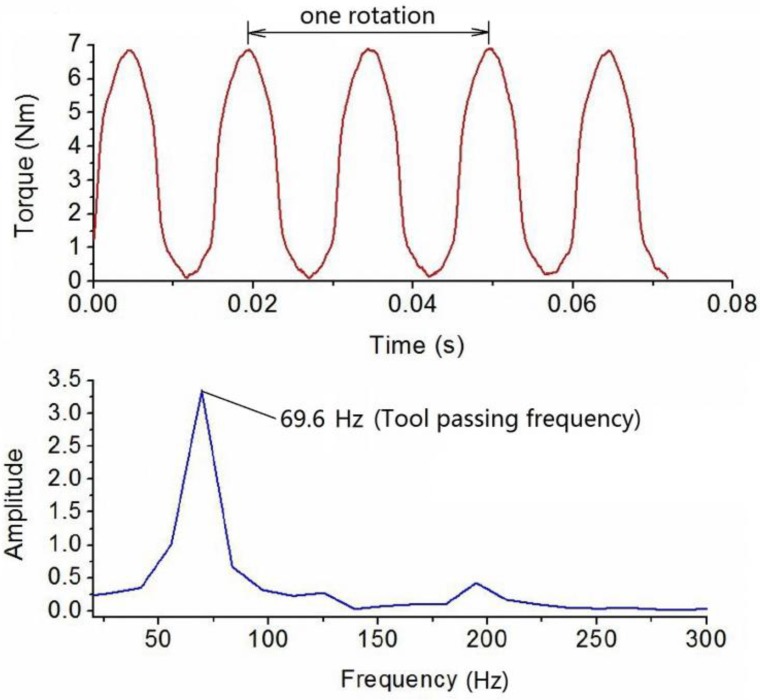
Plots of torque force signals in time and frequency domain at a spindle speed of 2000 rpm.

Dynamic cutting tests were performed in order to evaluate the performance of the developed sensor in real operation. The cutting tests were operated on a numerical control milling machine under dry cutting conditions as shown in Figure 11. The cutting tool was a two toothed 16 mm diameter end mill and the workpiece material was 7075 aluminum. These experiments were performed with a radial cutting depth of 100 percent immersion, axial cutting depth of 1 mm, feedrate of 0.15 mm per tooth under steady state milling conditions with all teeth in place. The spindle speed was separately set at 500, 1000 and 2000 rpm. During the experiments, the sampling rate of the cutting torque forces measurement was 10 kHz, and a pair of wireless signal processing and transmitter and receiver was also used. The acquired milling torque force signals are given in Figure 12, Figure 13 and Figure 14. The top graph shows the dynamic milling torque forces in the time domain. Their wave form in the frequncy domain is depicted in the bottom graph. All the spectra are respectively normalized by their own maximum values.

Figure 12 shows the results of measured milling torque forces with a spindle speed of 500 rpm, where the measured frequecy generated by the milling tool tooth is 17.2 Hz. Figure 13 and Figure 14 show the results that when the spindle speeds are increased to 1000 rpm and 2000 rpm, whereby the detected frequency also increased to 32.9 Hz and 69.6 Hz. During the milling process, because the cutting tool has two cutting teeth, the measured frequency derived from torque sensor approximately equals twice the spindle speed. All the spectra are normalized by the amplitude of the maximum frequency peak seen in the respective spectrum.

**Figure 15:**
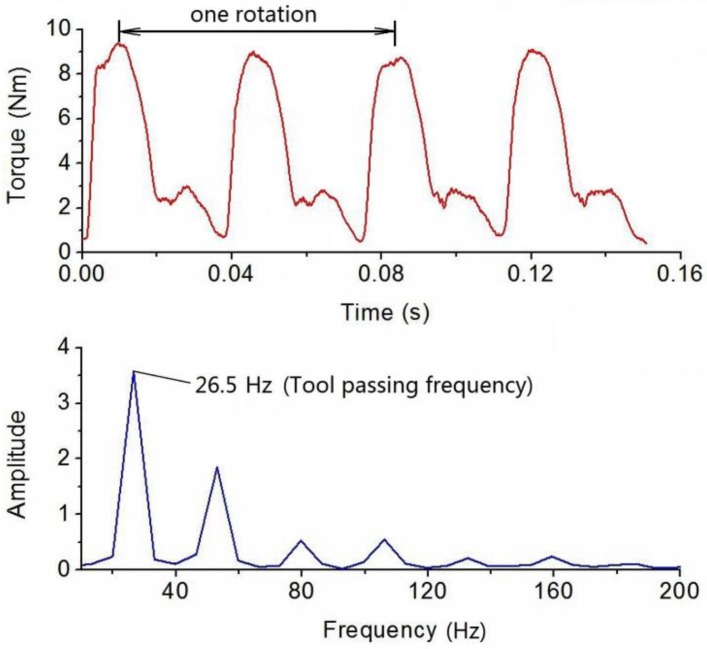
Plots of torque force signals in time and frequency domain at a spindle speed of 800 rpm.

A milling test record with the same milling parameters as mentioned above is presented in Figure 15, except that one of the milling tool teeth was broken and the rotation speed was 800 rpm. The irregular signal obviously indicates the effect of the broken milling tool tooth in the time domain. It means that the abnormal situation will be found immediately through the time domain when monitoring the machining process. Certainly, these frequencies have to be lower than a quarter of the natural frequency (1216 Hz). By comparing the measured milling torque signals, when the spindle speed increases with other milling parameters invariant, the measured torque value decreases slowly.

## 6. Conclusions

In this study, an innovative piezoresistive MEMS strain gauge-based torque sensor with both high sensitivity and high natural frequency was developed for milling processes. A piezoresistive MEMS stain gauge was employed to measure torque in milling processes for the first time. Cutting tools are interchangeable so the developed sensor can support a variety of machining processes. The static experiments showed that the sensitivity was approximately 0.13 mv/Nm, which is much higher than that of a traditional torque rotating dynamometer. The modal impacting test results show that the natural frequency of the sensor reaches 1216 Hz, which means that the dynamic operation range of the torque sensor is suitable for spindle speeds of less than 8445 rpm in machining processes when the cutting tool has two teeth. It is observed that the piezoresistive MEMS strain gauge-based torque sensor primely picks up the periodic frequency of the cutting tooth in the experiments. The experimental results indicate that the torque sensor based on a MEMS chip developed in this work optimizes the intrinsic contradiction between senstivity and natural frequency in traditional strain gauge sensors. It is feasible to use torque sensor monitoring of the stability of milling processes in real-time.

It is worth noting that the measured piezoresistive MEMS sensor signal is a slightly fluctuant version of the milling torque, especially the signal wave was anomalous when the milling tool was damaged as shown in Figure 15, so this variation can be used for monitoring the stability of machining processes under high speed cutting conditions. The variations of the measured signal could be used to reveal wear or breakage of the cutting teeth, to detect the occurrence of chatter, to monitor changes in cutting parameters such as feed rate, cutting depth, spindle rotation speed and so on. In conclusion, it would significantly benefit machining processes in monitoring and controlling applications.
